# Case Report: Two cases of multiples and atypical dermal sinus tracts

**DOI:** 10.3389/fped.2024.1346970

**Published:** 2024-01-22

**Authors:** Peter Spazzapan, Dominic N. P. Thompson

**Affiliations:** ^1^ Paediatric Neurosurgery Unit, Clinical Department of Neurosurgery, University Medical Centre Ljubljana, Ljubljana, Slovenia; ^2^ Department of Paediatric Neurosurgery, Great Ormond Street Hospital for Children, NHS Foundation Trust, London, United Kingdom

**Keywords:** dermal sinus tract, infection, case report, tethered cord, dysraphic malformation

## Abstract

Dermal sinus tracts (DSTs) are congenital lesions that connect the cutaneous ectoderm with the underlying neuroectodermal tissues. They are typically midline, solitary lesions. Multiple, and atypically located DSTs have been only rarely described. We present two cases of multiple and laterally located DSTs. The first presented with bacterial meningitis and two tracts in the right buttock, one of which entered the spinal canal through the S3 neural foramen. The second child had three midline lumbar DSTs, one subcutaneous dermoid cyst and one intradural epidermoid cyst. Complete surgical excision was achieved in both cases with good late follow up. Multiple or atypically located DSTs appear to carry the same risks of infection as the more common, midline, single tracts. Complete surgical excision is recommended to avoid the risks of neurological deterioration, in particular due to infection. Incomplete disjunction is the proposed developmental anomaly for DSTs, however the location of the cases presented here requires an alternative explanation.

## Introduction

Dermal sinus tracts (DSTs) are part of a spectrum of disorders that include limited dorsal myeloschisis (1). It is suggested that these represent a persisting connection between the cutaneous and neural derivatives of ectoderm through a process of incomplete disjunction. Usually, they occur in the midline and most frequently in the lumbar region (2). All dermal pits and dimples, which are not located within the intergluteal cleft, should be investigated in the early neonatal period, first with an ultrasound, to demonstrate the connection between the neural and the cutaneous structures. An MRI is then required, to confirm the diagnosis. Symptoms occur through various mechanisms that include tethering, dermoid cyst formation and infection. DSTs extend to variable depths below the skin: a minority end in extraspinal tissues, whilst the majority cross the dura and terminate above the conus (3–5). Inclusion tumors such as dermoids and epidermoids (6) coexist in 15%–43% of cases (3, 4, 7) and their natural history is that of slowly growing tumors that can reach large sizes and subsequently cause neurological deficits and pain. They can also act as a nidus for infection. Multiple and atipically located DSTs have been only rarely described and hereby we add two more cases to the very few described so far. One presented with two laterally located tracts in the right buttock, the other had three midline tracts in the lumbar spinal region.

## Case report

### Case 1

A two months old girl presented with fever and positive meningeal signs. She had otherwise no other pathology of malformation. At examination two small dimples in the gluteal region, overlying the right buttock were found. Both were located two centimetres lateral from the intergluteal fold.

**Figure 1:**
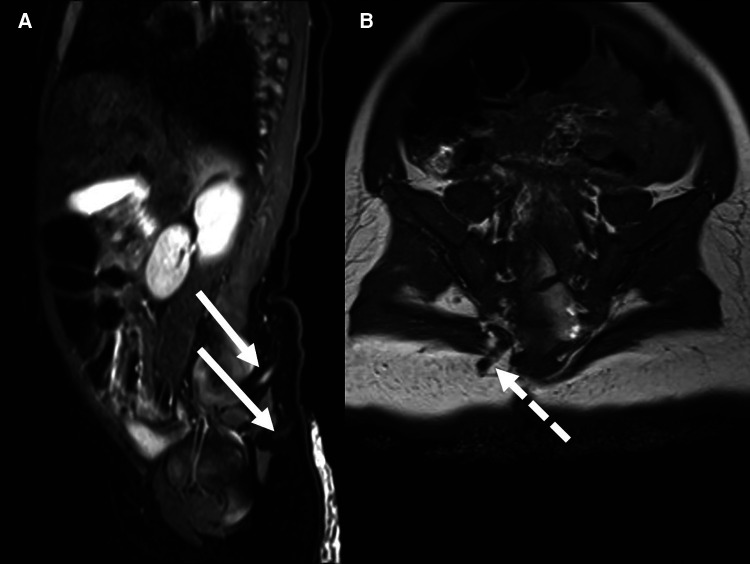
Mr in *case 1* revealed the presence of two tracts (*linear arrows*) passing from the skin of the right buttock medially toward the lower sacrum (**A**) the upper DST (**B**) entered the lower spinal canal through the S3 neural foramen (*dotted arrow)*. There were no radiological signs of intradural extension of the DST.

MR confirmed two DSTs passing through the subcutaneous tissues, with the upper tract terminating at the level of the lower sacrum at the level of the S3 neural foramen (Figure 1). There was no clear evidence of intraspinal extension. The conus appeared to lie at a normal level. There was no obvious evidence of intradural dermoid formation.

A first excision of the two DSTs was performed to the sacral bone, without performing a deeper intraspinal exploration of the upper tract. Histopathologic examination confirmed the diagnosis of two DSTs and found signs of local inflammation within the superior tract, which thus served as a corridor for the initial infective event. After one month, the child presented with an intermittent discharge from the wound, followed by the development of a superficial buttock abscess, that was excised and drained. Wound healing problems persisted and the child required a second procedure at which the residual tract was identified at the level of the neural foramen S3. The tract was followed medially and cranially by deroofing the posterior elements of the sacral spinal canal. Using the nerve root mapping it was possible to identify and preserve a large S3 nerve root, lying immediately below the line of the track, which was exposed for a length of 3 cm to its termination. Complete excision of the track was achieved. The postoperative course was uneventful, and no further complications have occurred within the next 4 years of follow up.

### Case 2

**Figure 2:**
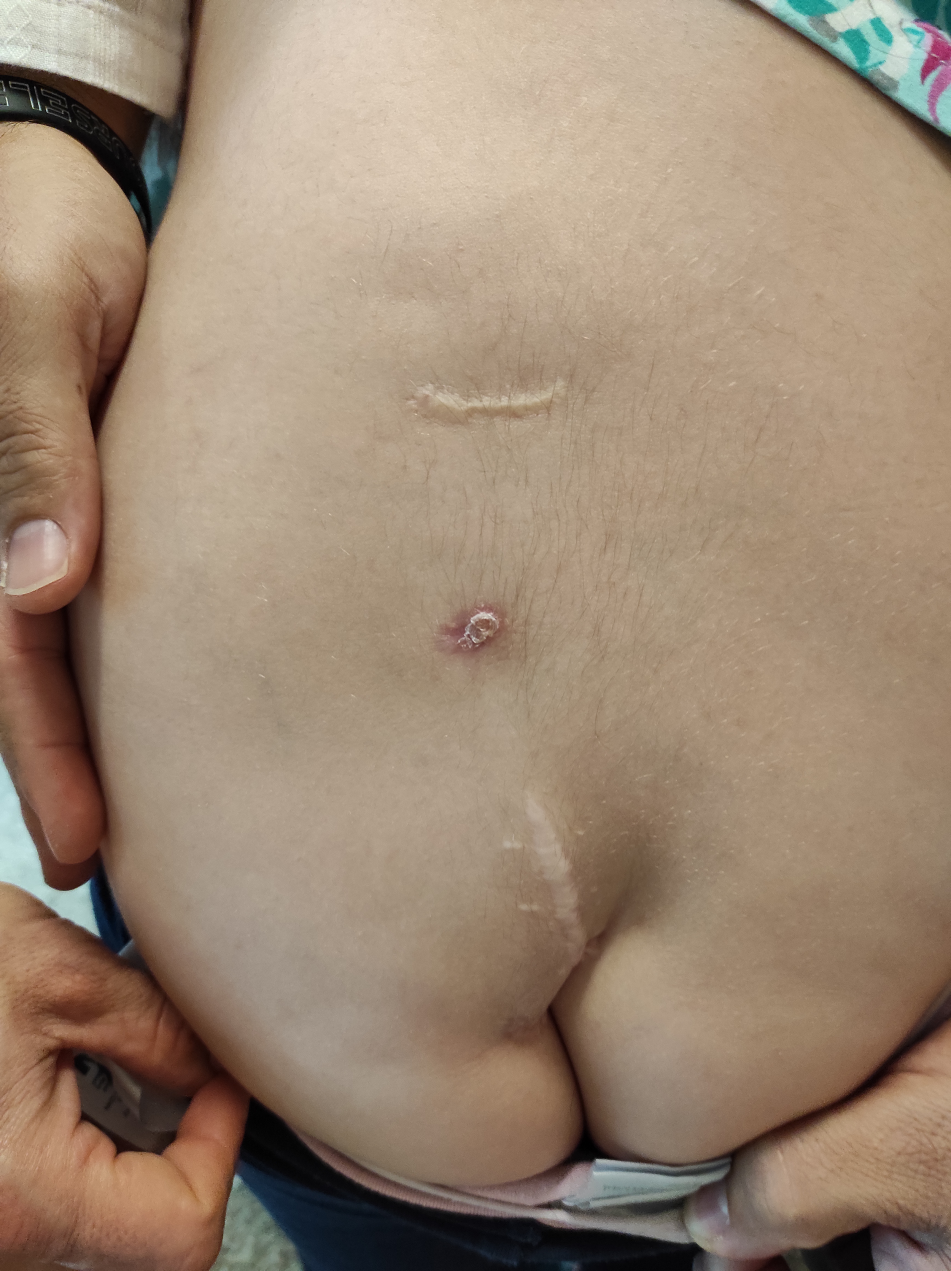
The child in *case 2* was followed from birth till the age of 7 years for three lumbar dimples, all located 1 to 2 cm left from the midline. Two of them have been characterized by recurrent discharge and two of them have been partially excised elsewhere. Despite this, no meningitis occurred.

A girl born with VACTERL association was operated soon after birth for a tracheoesophageal fistula. She had three cutaneous dimples in the lumbar region. All were paramedian, located at least 1 cm from the midline (Figure 2). Early in childhood, there was recurrent discharge from the two superior dimples. Superficial resection of these two lesions was undertaken elsewhere, when the child was one year old. She presented to neurosurgery at two years of age because of persistent discharge from the upper dimple.

**Figure 3:**
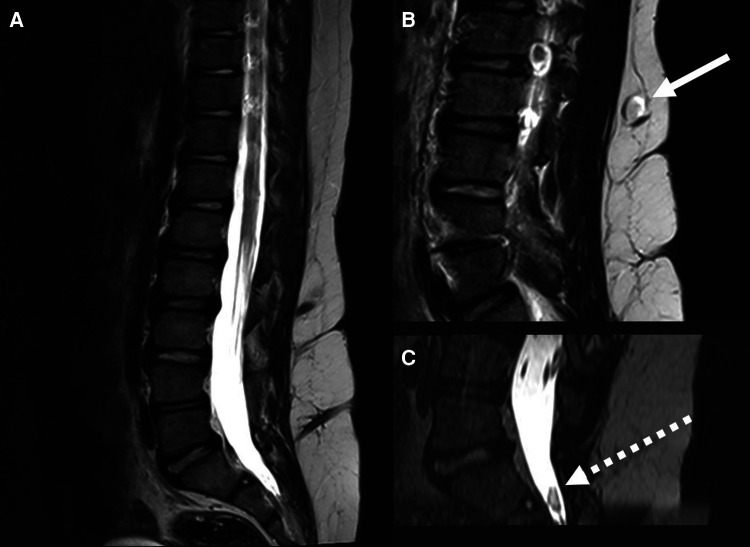
Mr in *case 2* revealed the presence of three DSTs, located at the levels L2, L3 and S1 (**A**) the conus was located at the level L2. The uppermost DST ended in a subcutaneous dermoid cyst (*linear arrow*) (**B**) an intradural inclusion cyst (*dotted arrow*) was visualized at the level S2 (**C**).

MRI revealed three DSTs (Figure 3A): one at level L2, ending in a subcutaneous inclusion cyst (Figure 3B), the second at level L3 and the third at level S1. There was no clear intradural extension of the tracts on MR, but an intradural inclusion cyst was recognized at level S2 (Figure 3C). The girl had a normal neurological examination, and the family declined neurosurgical treatment. Regular EMG were performed in the setting of a regular follow-up of children with occult spinal dysraphisms. At the age of seven years this examination revealed a mild deterioration of the motor function of the left lower limb. At that moment, the family agreed for surgery.

**Figure 4:**
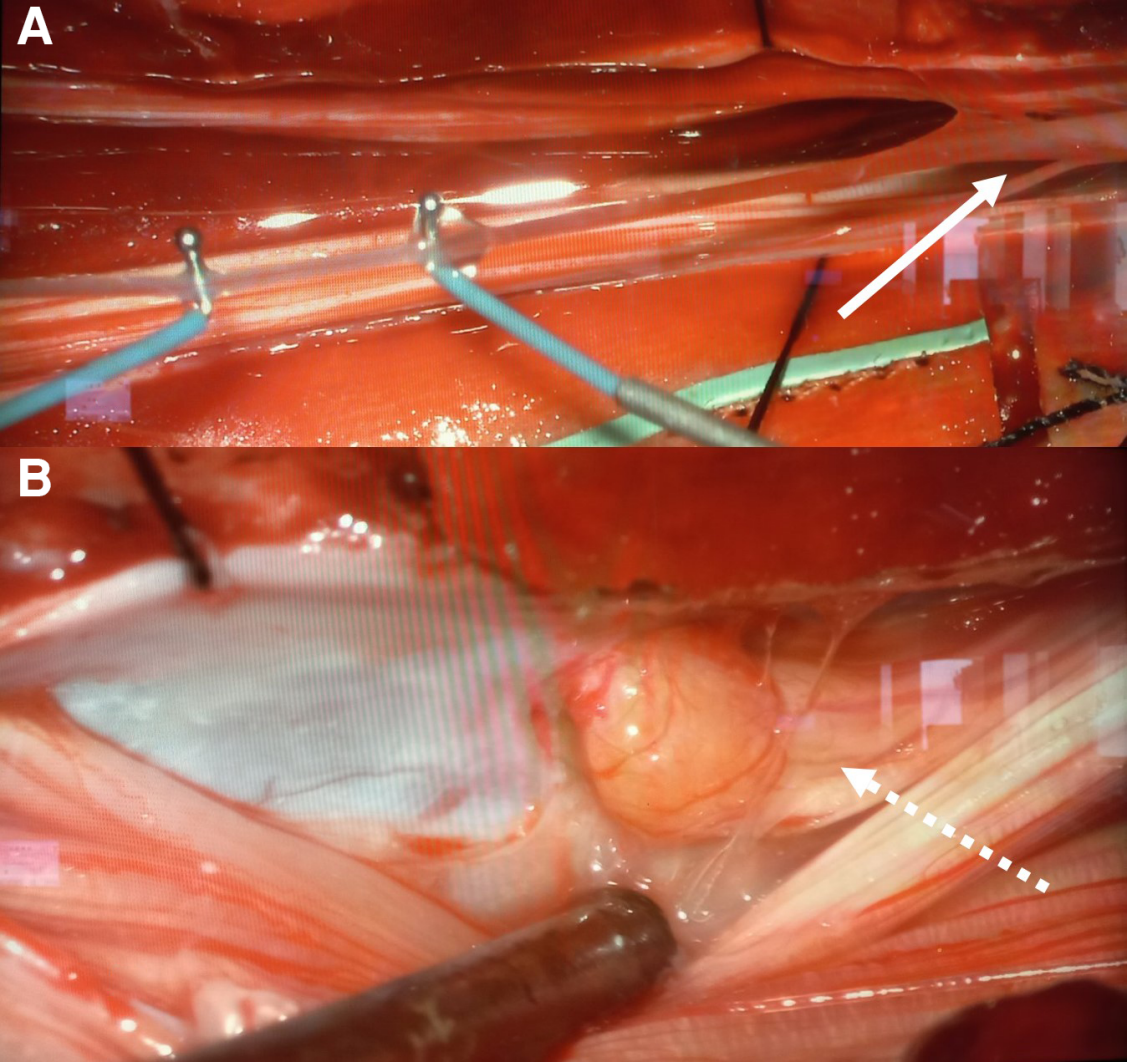
Intraoperatively a non-lipomatous filum terminale (*linear arrow*) (**A**) was found. The inclusion cyst was tightly attached to the left S2 root (*dotted arrow*) (**B**) a complete microsurgical removal was achieved by means of neuromonitoring.

At surgery, the three DSTs were followed within the subcutaneous tissue until the level of the spinous processes. A laminotomy was performed from L2 to S2. At the elevation of the laminae no tract was visualized in direct contact with the dorsal dural surface. A wide intradural exploration was performed but no clear DST was visualized. The filum terminale was followed in a caudal direction (Figure 4A) to the level S2, where the epidermoid cyst was found. This was attached to the dorsal aspect of the left S2 root (Figure 4B). Complete microsurgical excision was achieved by means of intraoperative neuromonitoring. After dural closure, the laminae were fixed with resorbable plates. The postoperative course was uneventful. Histological examination confirmed the diagnosis of three subcutaneous DSTs and of one extraspinal dermoid cyst, in which the uppermost tract terminated. Furthermore, histopathological exams revealed the diagnosis of intradural epidermoid cyst and the absence of an intradural DST.

## Timeline

### Case 1


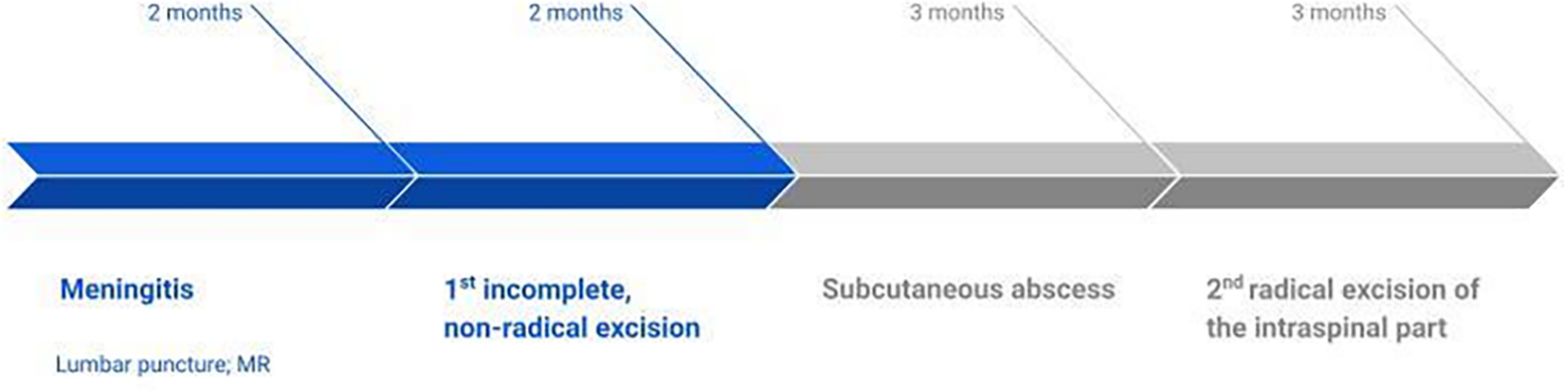


### Case 2


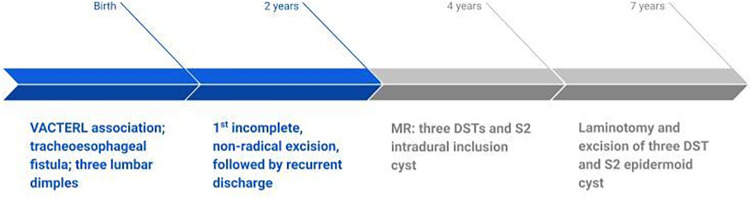


## Discussion

DSTs are a form of dysraphic lesions that result from an incomplete disjunction between neural and cutaneous ectoderm. DSTs are usually single lesions and only few reports describe multiple and atypically located DSTs (2, 8–12). Rarely, they can also be associated to other malformations, such as a cystic dilatation of ventriculus terminalis, a malformation known to develop during various stages of vacuolisation, canalisation and retrogressive differentiation (13). Double DSTs along the spine have been described in two cases: one case of double, midline DTSs in the cervical region (2) and one case of double midline DSTs with cervico-thoracic location (12). A case of triple lumbar DST has been reported only in one case (8), in which two paramedian lumbar tracts ended into two separate extraspinal dermoid tumors, while the third tract, located in the midline, above the intergluteal cleft terminated in an intradural dermoid tumor. Atypically located DSTs have also been described only in few reports (9, 11). Carillo et al. reported two cases, both in the buttock region and both with intraspinal extension. One child in this report had a dimple at the left buttock that penetrated through the ligament and reached the dura, though there were no intradural abnormalities. The second child had three dimples on her lower back: one was located in the left buttock and communicated with a subcutaneous and epidural lipoma. In the intradural space there were only small amounts of fat tissue (9). Last, Inwueke (11) described a child with a dimple on her left buttock that ended blindly in the subcutaneous tissue.

DSTs constitute a potential conduit for the passage of microorganisms, therefore meningitis or abscess may occur at any time. The risk of infection or abscess formation seems to be more likely when there has been a history of discharge from the cutaneous orifice and in the presence of a spinal inclusion cyst (7). Neurological examination at presentation is most frequently normal (3) and neurological deterioration can be related to infective complications, to the mass effect of an inclusion cyst and to spinal cord tethering (3, 4, 6).

It is generally recommended that an early prophylactic surgery with the use of intraoperative neuromonitoring is recommended for all DSTs, as the surgical risk is low and the risk of infective complications, in untreated DSTs, remains high (4, 6). A neurosurgical consultation should be obtained as soon as the diagnosis of DST is established and performing surgery without a neurosurgical expertise should be always avoided. Complete visualization of the tract, including intradural exploration, is indicated even in the absence of visible intradural pathology on MRI (14). The surgical treatment of DSTs is even more imperative in cases presenting with a history of discharge or in those harbouring an inclusion cyst. An incomplete excision can result in acute or chronic infective complications (14), as demonstrated by our two presented cases. Whenever an inclusion cyst is not totally excised, remnants of the cyst wall may represent a nidus for recurrence (15). Despite this, most series report surprisingly low rates of recurrence even after incomplete removal of the tracts and inclusion cysts. The long-term outcomes are generally good (15, 16) and clearly better outcomes are reported where surgery has been performed in uncomplicated conditions. In particular, preoperative infective complications can negatively affect the prognosis. If the child does develop infectious or meningeal signs before surgical treatment, a targeted antibiotic therapy should be initiated, until complete recovery of the infective complication. After that, an MRI should be repeated and a delayed excision of the DST should be planned.

The embryogenetic mechanism for these lesions remains unclear. Regarding multiple DSTs a single midline tract might occur after a failure during disjunction, followed by a later, second event causing its separation in two or more tracts (8). Another hypothesis is that the multiple tracts might arise at different stages of the embryogenesis. Those that do not penetrate the dura might develop only after the disjunction process, at a stage when the normal formation of mesenchymal layers has been already concluded. On the other hand, laterally located DSTs at the level of the buttock should be explained by abnormalities during secondary neurulation. Recent studies in avian embryos have shown that there is a heterogeneity of cells within the region of the tail bud. These cells exhibit a restriction of developmental potentials: the rostral cells generate neural elements of the distal spinal cord, the intermediate cells display both neural and mesodermal potentials, while the most caudal cells give rise to mesodermal elements (17). Neural elements in this region are formed through the phenomenon of ingression, this is by an epithelial-mesenchymal transition. The origin of DSTs during secondary neurulation could be explained by abnormalities of the extreme caudal part of the tail bud and by an abnormal ingression process of the superficial neural tissues.

## Data Availability

The original contributions presented in the study are included in the article/Supplementary Material, further inquiries can be directed to the corresponding author.

## References

[1] Pang D Zovickian J Oviedo A Moes GS. Limited dorsal myeloschisis: a distinctive clinicopathological entity. Neurosurgery. (2010) 67:1555–79. discussion 1579–80. 10.1227/NEU.0b013e3181f93e5a 21107187

[2] Khashab M E Nejat F E. A double dermal sinuses: a case study. J Med Case Rep. (2008) 2(1):281. 10.1186/1752-1947-2-281 18727820 PMC2531124

[3] Radmanesh F Nejat F El Khashab M. Dermal sinus tract of the spine. Childs Nerv Syst. (2010) 26:349–57. 10.1007/s00381-009-0962-z 19662426

[4] Ackerman LL Meneses AH. Spinal congenital dermal sinuses: a 30 year experience. Pediatrics. (2003) 112:641–7. 10.1542/peds.112.3.641 12949296

[5] Tubbs RS Frykman PK Harmon CM Oakes WJ Wellons JC. An unusual sequelae of an infected persistent dermal sinus tract. Childs Nerv Syst. (2007) 23:569–71. 10.1007/s00381-006-0216-2 16944171

[6] Kanev PM Park TS. Dermoids and dermal sinus tracts of the spine. Neurosurg Clin N Am. (1995) 6(2):359–66. 7620359

[7] Thompson DNP. Spinal inclusion cysts. Childs Nerv Syst. (2013) 29:1647–55. 10.1007/s00381-013-2147-z 24013335

[8] Ansari S Andrabi Y El Khashab M Bateni F Dadmehr M Iskandar BJ Triple lumbar dermal sinuses: unusual presentation of a typically solitary midline lesion. Pediatr Neurosurg. (2009) 45(4):305–7. 10.1159/000235605 19690447

[9] Carrillo R Carreira LM Prada JJ Rosas C. Lateral congenital spinal dermal sinus. Child’s Nerv Syst. (1985) 1:238–40. 10.1007/BF00270770 3905000

[10] Nejat F Dias MS Eftekhar B Nasri Roodsari N Hamidi S. Bilateral retro-auricular dermal sinus tracts with intradural extension. J Neurosurg. (2003) 99:163–6. 10.3171/jns.2003.99.1.0163 12854760

[11] Ikwueke I Bandara S Fishman SJ Vargas SO. Congenital dermal sinus tract in the lateral buttock: unusual presentation of a typically midline lesion. J Pediatr Surg. (2008) 43:1200–2. 10.1016/j.jpedsurg.2008.01.021 18558207

[12] Mrowczynski OD Lane JR Shoja MM Specht CS Langan ST Rizk EB. Double dermal sinus tracts of the cervical and thoracic regions: a case in a 3-year-old child and review of the literature. Childs Nerv Syst. (2018) 34(5):987–90. 10.1007/s00381-017-3707-4 29279962

[13] Ganau M Talacchi A Cecchi PC Ghimenton C Gerosa M Faccioli F. Cystic dilation of the ventriculus terminalis. J Neurosurg Spine. (2012) 17(1):86–92. 10.3171/2012.4.SPINE11504 22559279

[14] Tisdall MM Hayward RD Thompson DN. Congenital spinal dermal tract: how accurate is clinical and radiological evaluation? J Neurosurg Pediatr. (2015) 15(6):651–6. 10.3171/2014.11.PEDS14341 26030333

[15] Fleming C Kaliaperumal C O’Sullivan M. Recurrent intramedullary epidermoid cyst of conus medullaris. BMJ Case Rep. (2011) 13:2011. 10.1136/bcr.11.2011.5090 PMC3238133 22669964

[16] Lunardi P Missori P Gagliardi FM Fortuna A. Long-term results of the surgical treatment of spinal dermoid and epidermoid tumors. Neurosurgery. (2008) 25:860–4. 10.1227/00006123-198912000-00002 2601815

[17] Catala M. Overview of secondary neurulation. J Korean Neurosurg Soc. (2021) 64(3):346–58. 10.3340/jkns.2020.0362 33906344 PMC8128529

